# Human Cardiac-Derived Stem/Progenitor Cells Fine-Tune Monocyte-Derived Descendants Activities toward Cardiac Repair

**DOI:** 10.3389/fimmu.2017.01413

**Published:** 2017-10-26

**Authors:** Noémie Dam, Hocine Rachid Hocine, Itziar Palacios, Olga DelaRosa, Ramón Menta, Dominique Charron, Armand Bensussan, Hicham El Costa, Nabila Jabrane-Ferrat, Wilfried Dalemans, Eleuterio Lombardo, Reem Al-Daccak

**Affiliations:** ^1^Coretherapix SLU, Tigenix Group, Madrid, Spain; ^2^Institut National de la Santé et de la Recherche Médicale (INSERM) UMRS-976, Université Paris-Diderot, Hôpital Saint-Louis, Paris, France; ^3^Tigenix, Madrid, Spain; ^4^HLA et Médecine, Hôpital Saint Louis, Paris, France; ^5^Centre National de la Recherche Scientifique (CNRS), Centre of Pathophysiology Toulouse Purpan, INSERM, Université Toulouse III, CHU Purpan, Toulouse, France; ^6^Tigenix NV, Leuven, Belgium

**Keywords:** monocytes, macrophages, dendritic cells, cardiac-derived stem/progenitor cells, allogeneic stem cells therapy

## Abstract

Cardiac repair following MI relies on a finely regulated immune response involving sequential recruitment of monocytes to the injured tissue. Monocyte-derived cells are also critical for tissue homeostasis and healing process. Our previous findings demonstrated the interaction of T and natural killer cells with allogeneic human cardiac-derived stem/progenitor cells (hCPC) and suggested their beneficial effect in the context of cardiac repair. Therefore, we investigated here whether monocytes and their descendants could be also modulated by allogeneic hCPC toward a repair/anti-inflammatory phenotype. Through experimental *in vitro* assays, we assessed the impact of allogeneic hCPC on the recruitment, functions and differentiation of monocytes. We found that allogeneic hCPC at steady state or under inflammatory conditions can incite CCL-2/CCR2-dependent recruitment of circulating CD14^+^CD16^−^ monocytes and fine-tune their activation toward an anti-inflammatory profile. Allogeneic hCPC also promoted CD14^+^CD16^−^ monocyte polarization into anti-inflammatory/immune-regulatory macrophages with high phagocytic capacity and IL10 secretion. Moreover, hCPC bended the differentiation of CD14^+^CD16^−^ monocytes to dendritic cells (DCs) toward anti-inflammatory macrophage-like features and impaired their antigen-presenting function in favor of immune-modulation. Collectively, our results demonstrate that allogeneic hCPC could reshape monocytes, macrophages as well as DCs responses by favoring their anti-inflammatory/tolerogenic activation/polarization. Thereby, therapeutic allogeneic hCPC might also contribute to post-infarct myocardial healing by modeling the activities of monocytes and their derived descendants.

## Introduction

Since their discovery (1), human adult cardiac stem/progenitor cells have become a valuable source for the development of autologous and allogeneic cardiac regenerative/reparative therapies (2–5). Because autologous strategies have encountered certain limitations, the new era tends to acknowledge allogeneic stem/progenitor cells as being a more realistic and pragmatic cardiac repair strategy (4).

Recent progress has provided new insights into the mode of action and immune behavior of these stem/progenitor cells within autologous and allogeneic settings. It is very likely that they repair the injured myocardium through constructive paracrine rather than trans-differentiation mechanisms (6, 7). This applies to mesenchymal stem cells, cardiosphere-derived cells (CDC) and cardiac-derived stem/progenitor cells (CPC) (8–10).

Human CPC (hCPC) are cardiac-derived progenitor cells with a mixed stem-cell phenotype (11). They express SSEA-1, SSEA-4, CD90, CD73, CD105, and CD166 stem/progenitor markers and low levels of c-Kit. They also express the pluripotency transcription factors OCT4, SOX2, and NANOG and are negative for hematopoietic and endothelial cell markers. hCPC express the cardiac lineage commitment factors Nkx2.5, GATA-4, Islet-1, and MEF2C. Immunologically, they express human leukocyte antigens (HLA) class I molecules but negligible levels of HLA class II, are negative for co-stimulatory molecules CD40, CD80, and CD86, but express PD-L1 (CD274). The presence of hCPC within an inflammatory environment, without modifying their stem/progenitor phenotype changes their immunological profile notably upregulates expression of HLA molecules. These cells differentiate into the three principal cardiac lineages *in vitro* and promote cardiac repair/regeneration in experimental mouse models (11). Our previous findings on hCPC highlighted their allogenicity as part of the constructive paracrine mechanisms that could mediate the therapeutic effects of hCPC (11–14). With this promising preclinical background, hCPC are currently under clinical investigation in the ongoing double-blind, 2:1 randomized, controlled, and multicenter clinical trial ongoing Cardiac Stem Cells in Patients With Acute Myocardial Infarction (CAREMI) phase I/II clinical trial, with detailed safety immunologic assessments and magnetic resonance imaging-based efficacy end points (https://clinicaltrials.gov/ct2/show/NCT02439398) (15).

Cardiac repair following MI relies on a finely regulated immune response. One of the crucial steps required for successful restoration of tissue homeostasis depends on the sequential recruitment of circulating monocytes to the injured tissue (16, 17). These cells are characterized by a considerable plasticity and depending on their microenvironment, they give rise to different phagocytic populations, including macrophages and dendritic cells (DC) (18). The monocytes-derived cells are also at play upon MI through the initiation and resolution of inflammation, phagocytosis, proteolysis, angiogenesis, infarct healing, and ventricular remodeling (19–23).

The cytokines, macrophage colony-stimulating factor (M-CSF), and granulocyte-macrophage colony-stimulating factor (GM-CSF) are the main factors driving differentiation of monocytes into macrophages. Additional stimuli lead to distinct macrophage subsets with unique phenotypes and functional abilities. For instance, toll-like receptor (TLR) activation and the presence of interferonγ (IFNγ) will lead to their differentiation into classically activated pro-inflammatory M1 macrophages. In contrast, interleukin 4 (IL4), IL13, and IL10 trigger macrophage polarization toward an alternatively activated anti-inflammatory, immune-regulatory, or tissue remodeling M2 (M2a, M2b, and M2c, respectively) phenotype (24–26). Monocytes have also the potential to differentiate into professional antigen-presenting cells, the myeloid DC with overlapping functions and phenotype (27, 28). The presence of GM-CSF and IL4 promotes blood monocytes differentiation into immature DC, which can further differentiate into mature DC (mDC) upon activation to initiate the adaptive immune response (28, 29).

A key challenge to promoting allogeneic hCPC cells to the level of efficient successful clinical practice is deciphering their projected immune-mediated paracrine regenerative/reparative action. Since monocytes and their descendants are crucial for cardiac repair following MI, we investigated whether these cells are involved in the immunomodulatory/reparative action of allogeneic hCPC. Through experimental *in vitro* assays, we assessed the impact of allogeneic hCPC on the recruitment, functions, and differentiation of monocytes. Our results show that allogeneic hCPC at steady state or under inflammatory conditions can incite CCL-2/CCR2-dependent recruitment of circulating CD14^+^CD16^−^ monocytes and fine-tune their activation/polarization toward an anti-inflammatory profile. Moreover, hCPC promote monocyte differentiation into anti-inflammatory/immune-regulatory macrophages with high phagocytic capacity and IL10 secretion. Finally, hCPC bended the differentiation of monocytes to DC toward anti-inflammatory/immune-modulatory macrophage-like features and impaired their function. Thereby, therapeutic allogeneic hCPC might contribute to post-infarct myocardial healing by modeling the monocyte-derived cell functions.

## Materials and Methods

### Ethical

The study was approved by the local research ethical committee and “Comité consultatif pour la protection des personnes dans les recherches biomédicales.” All experiments were performed in accordance with the local institutional guidelines and regulations and the approval of the local ethic committee, and with informed consent from all subjects. Human cardiac biopsies were obtained from patients undergoing open-chest surgery after signed informed consent in accordance with the Declaration of Helsinki. The ethical committees of “Hospital 12 de Octubre” and “Fundación Jiménez Díaz” (Madrid), Spain have approved the project. Blood donors signed an informed consent following human ethics committee from the “Centro de transfusion de la Comunidad de Madrid” and the institution has approved the study.

### Isolation and Culture of hCPC

Human CPC were obtained from the right atria appendage. Briefly, biopsies were minced and digested with collagenase type 2. Cell suspensions were centrifuged, filtrated with 45 µm strainers then hCPC were obtained by immunodepletion of CD45-positive cells followed by immunoselection of CD117 (c-kit)-positive cells, using specific microbead kits (Miltenyi Biotec, Madrid, Spain). All experiments were performed at 3% O2 with passage 4 and 7 HLA-typed hCPC and IFNγ-hCPC, mimicking those primed/stimulated by inflammatory environment (11), at 80–90% confluence and reproduced in at least three independent experiments with the same hCPC donor. Experiments were conducted with hCPC from three different donors. IFNγ-hCPC were washed before co-cultures with monocytes or their descendant cells to avoid any stimulation by residual IFNγ.

### Activation/Polarization and Differentiation of CD14^+^CD16^−^ Monocytes Cells and Co-Cultures with hCPC

Peripheral blood mononuclear cells (PBMC) were prepared from blood samples of healthy donors (*n* = 10). PBMC were isolated from buffy coats by Ficoll-Paque Plus density gradient (GE-Healthcare) centrifugation. CD14^+^CD16^−^ monocytes were isolated from the PBMC by magnetic immune-depletion using Dynabeads untouched human monocytes kit (Life Technologies, Madrid, Spain), following manufacturer’s instructions. Purity was 95%. CD14^+^CD16^−^ monocytes were activated with a combination of 50 ng/mL human recombinant M-CSF (Peprotech, bioNova cientifica, Madrid, Spain), 20 ng/mL lipopolysaccharide (LPS) (Sigma-Aldrich) and 100 U/mL IFNγ (eBioscience, Madrid, Spain) or with a combination of 50 ng/mL M-CSF, 20 ng/mL IL4 and IL13 (Peprotech). The differentiation of CD14^+^CD16^−^ monocytes toward DC was induced with 5 ng/mL human recombinant GM-CSF and 10 ng/mL IL4 (Peprotech) treatment for 5 days. In some experiments, 40 ng/mL LPS were added at day 5 for another couple of days to induce the maturation of DC. Monocyte cultures and co-cultures with hCPC or IFNγ-hCPC at a monocyte/hCPC ratio of 5:1 were conducted in RPMI 1640 medium supplemented with 10% FBS, 2mM l-glutamine, 100 U/mL and 100 µg/mL penicillin–streptomycin for 5 days in 6-well plates. In some experiments, co-cultures were performed in transwell chambers with 0.4 µm pores where hCPC or IFNγ-hCPC were seeded in the upper compartment.

### Immune Phenotyping

The expression level of relevant markers of CD14^+^CD16^−^ monocytes and their descendants’ under various culturing systems was determined by flow cytometry using specific antibodies. Briefly, cells were incubated with saturating concentration of human serum for 10 min at 4°C to prevent unspecific binding to Fcγ receptors and stained with mouse anti-CD45 FITC, anti-CD14 APC, anti-CD1a PE, anti-CD16 PE, anti-CD32 PE, anti-CD40 PE, anti-CD80 PE, anti-CD86 PE, anti-CD83 PE, anti-CD163 PE, anti-CD206 PE, anti-Toll-like receptor type 2 (TLR2) PE (BD Biosciences, Madrid, Spain), anti-HLA class II PE (eBioscience), and anti-CD64 PE (Life Technologies) monoclonal antibodies (Table S1 in Supplementary Material) for 30 min at 4°C. Cells were washed and then stained with 7-aminoactinomycin D (7-AAD) to exclude dead cells. Matched isotypes were used as negative controls. Cells were acquired using BD Fortessa flow cytometer (BD Biosciences) and analyzed using FACSDiva 8 (BD Biosciences) and FCS Express 6 (*De Novo* software, CA, USA) software. The expression of various markers is presented as the percentage of positive cells (%) and geometric mean fluorescence (Geometric mean).

### Transwell Migration Assay

Migration of CD14^+^CD16^−^ monocytes was determined by flow cytometry in Boyden chambers with 5 µm pores. hCPC and IFNγ-hCPC were cultured in RPMI medium for 24 h then cell-free conditioned media were collected and used as stimuli in the lower compartment of the transwell chambers. Freshly isolated CD14^+^CD16^−^ monocytes were plated in the upper compartment. The number of migrated monocytes (into the lower compartment) was counted by flow cytometry using a fixed sample volume and a fixed acquisition time (1 min). To normalize and standardize the counts a fixed amount of fluorescent microbeads of known concentration (Flow Cytometry Absolute Count Standard Beads, Immunostep, Spain) were used. The migration index was calculated as follow: number of cells migrated in presence of a stimulus/number of cells migrated in absence of stimulus. In another series of experiments, the monocytes were pre-incubated or not with 20 nM INCB 3284 dimesylate (Sigma-Aldrich, Madrid, Spain), a CCR-2 antagonist, or hCPC and IFNγ-hCPC conditioned media were incubated with 5 µg/mL polyclonal anti-CCL-2 or anti-IgG (Peprotech) prior to migration assays.

### Phagocytosis Assay

The phagocytic activity of CD14^+^CD16^−^ monocytes or their descendants derived at various culturing conditions, as indicated, was measured as the cellular uptake of zymosan A bioparticles. After co-culture with hCPC or IFNγ-hCPC, cells were collected and incubated with 0.5 mg/mL Red pHrodo Zymosan A BioParticles conjugate (Life Technologies) in RPMI medium for 90 min at 37°C. Cells incubated with red pHrodo zymosan A bioparticles at 4°C were used as negative controls. To stop the phagocytosis and remove the excess of red pHrodo zymosan A bioparticles, cells were washed in cold PBS 1× then stained with mouse anti-human CD45-FITC and 7-AAD. The phagocytic activity is evaluated by flow cytometry as the % pHrodo-positive cells quantified on 7-AAD-negative CD45-positive cells.

### Immunomodulation Assay

Peripheral blood mononuclear cells were depleted of CD14-positive cells [peripheral blood lymphocytes (PBLs)] using MACS CD14 microbeads (Miltenyi Biotec) then PBLs were stained with 10 µM carboxyfluorescein succinimidyl ester (CFSE, Sigma Aldrich) for 10 min. Within autologous settings, CFSE-labeled PBLs were stimulated with the T cell polyclonal activator PHA (0.5 µg/mL) in the presence or absence of CD14^+^CD16^−^ monocytes descendants, derived in 0,4 µm polycarbonate transwell chambers in 6-well plates (Corning) under various culturing conditions with allogenic hCPC, at 300,000 PBLs per 60,000 monocytes (5:1 ratio). T cells proliferation was quantified after 5 days by analyzing CFSE intensity on 7-AAD-negative CD3-positive cells using flow cytometry. The division index, defined as the average number of cells that a dividing cell became, was calculated using FCS express 6 software (*DeNovo* software).

### T Cells Proliferation Assay

The capacity of immature or mDC to stimulate an allogeneic T cells response was determined by mixed lymphocyte reaction assay. Briefly, immature or mDC derived in the presence or absence of hCPC, were co-cultured with allogeneic CFSE-labeled PBLs in 6-well plates (Falcon, Cat. 353046) for 5 days at 300,000 PBLs per 60,000 DC (ratio5:1). Proliferation of CD4^+^, CD8^+^ T cell subsets was monitored by flow cytometry using anti-CD3-APC, anti-CD4-Pacific blue, anti-CD8-APC-H7 (BD Biosciences), anti-CD25-PE (Miltenyi Biotec), and 7-AAD.

### Cytokine Analysis

The levels of CCL-2 and CX_3_CL-1 in hCPC and IFNγ-hCPC supernatants and of IL10 and TNF-α in supernatants obtained from various co-culture settings were determined by enzyme-linked immunosorbent assay (ELISA) using specific kits (R&D Systems, Biogen cientifica, Madrid, Spain) following manufacturer’s instructions. Each analysis was performed at least in duplicate. When co-culturing experiments were conducted in transwell chambers, CD14^+^CD16^−^ monocytes or their descendants derived at various culturing conditions were collected, washed, and cultured alone for another 48 h. Supernatants were then collected for quantification of IL10 and TNFα.

### Statistical Analysis

Statistical analysis was performed using GraphPad Prism 7 (GraphPad Software Inc.). Descriptive data were presented as mean ± SEM from at least four independent experiments conducted with PBMC isolated from different donors against the same hCPC. Comparison between conditions was performed using one-way ANOVA multiple comparisons and Bonferroni’s multiple comparison *post hoc* test. A *p*-value of less than 0.05 was considered statistically significant. *P < 0.05; **P < 0.01; ***P < 0.001; ****P < 0.0001. When stated in the figure legend, each data point represent the mean of experimental triplicates.

## Results

### Human CPC Support MCP-1/CCR2-Dependent Recruitment of Monocytes

**Figure 1:**
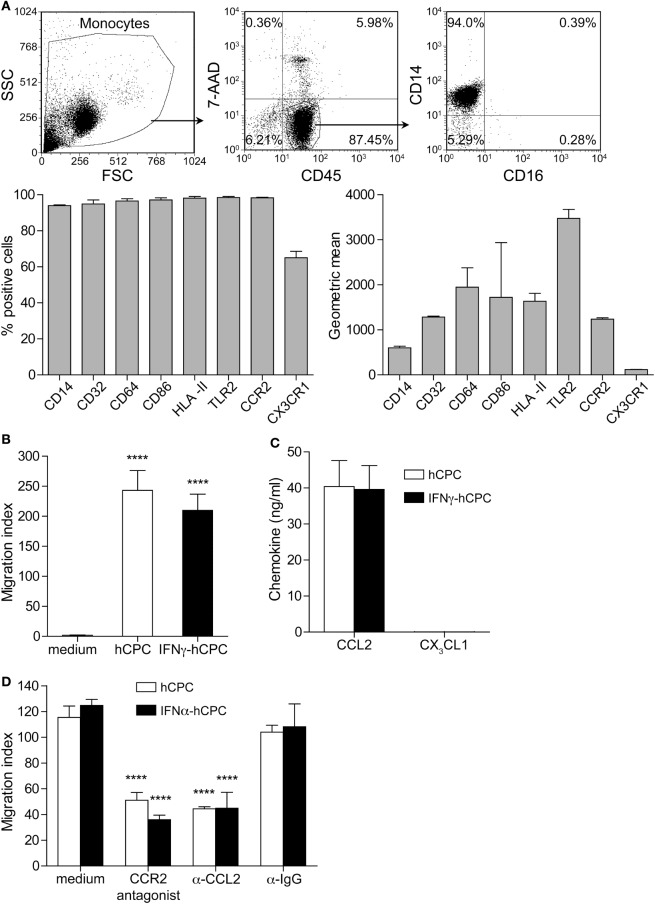
Human cardiac-derived stem/progenitor cells (hCPC) have the capacity to recruit CD14^+^CD16^−^ monocytes. **(A)** Expression of informative markers on CD14^+^CD16^−^ monocytes isolated from peripheral blood mononuclear cells (PBMC) as determined by flow cytometry (representative dot plot in the upper panel). Results are presented as percentage of positive cells (lower left panel) and geometric mean (lower right panel). **(B)** Migration of monocytes in response to hCPC- or interferonγ (IFNγ)-hCPC conditioned media or to RPMI medium was determined by transwell migration assay. The number of migrating cells was determined using flow cytometry counting beads. **(C)** The levels of CCL2 and CX_3_CL1 produced by hCPC- and IFNγ-hCPC as determined by specific enzyme-linked immunosorbent assay. **(D)** CD14^+^CD16^−^ monocytes migration in response to hCPC and IFNγ-hCPC conditioned medium in the presence of 20 nM INCB 3284 dimesylate CCR-2 antagonist, 5 µg/mL polyclonal anti-MCP-1, or anti-IgG isotype control was determined as in **(B)**. Data on the graphs represent the mean ± SEM from five independent experiments conducted with PBMC isolated from five different donors against the same hCPC, and are representative of data obtained with two other hCPC from different donors. Each data point represents the mean of experimental triplicates. ****P < 0.0001.

Monocytes and their cellular derivatives play a crucial role in tissue repair either directly or via paracrine secreted factors. Human CD14^+^CD16^−^ monocytes are phagocytic effectors (18) that express CD14, FcγRI CD64 and FcγRII CD32, major histocompatibility complex class II molecules (HLA II in humans), CD86 co-stimulatory molecule, and toll-like-receptor 2 (TLR-2) at variable levels (Figure 1A). They lack the expression of FcγRI CD16, scavenger receptor CD163 and mannose receptor CD206.

To understand whether monocytes and their descendants may contribute to hCPC-instructed cardiac repair, we first checked whether hCPC could attract blood CD14^+^CD16^−^ monocytes in allogeneic setting. In theory, the infused hCPC to MI patients would operate within an inflammatory environment rich in a variety of growth factors and pro-inflammatory cytokines, such as IFNγ and TNFα, which would change their immunological/inflammatory profile without affecting their stem/progenitor properties (11). Thus, hCPC were used at steady state and after treatment with IFNγ (IFNγ-hCPC) to mimic MI inflammatory context. We quantified the migration of freshly isolated CD14^+^CD16^−^ monocytes (Figure 1A) in response to conditioned media from hCPC- or IFNγ-hCPC using Boyden chambers. Under these settings, monocytes significantly migrated in response to both hCPC- and IFNγ-hCPC conditioned media, yielding comparable migration index (Figure 1B).

Human blood monocytes subsets express various chemokine receptors that govern their specific trafficking (18). Freshly isolated CD14^+^CD16^−^ monocytes express CCR2 (CCL2 receptor) and at a lower level CX_3_CR1 (CX_3_CL1 receptor) (Figure 1A; Figure S1A in Supplementary Material) but migrated only in response to increasing concentrations of recombinant human CCL2 (Figure S1B in Supplementary Material). hCPC and IFNγ-hCPC produce substantial amounts of CCL2 (around 40.0 ± 7.2 ng/mL) but do not secret any detectable amounts of CX_3_CL1 (Figure 1C). Accordingly, we examined effects of CCR-2 antagonist INCB 3284 dimesylate and neutralizing anti-CCL2 antibody on the migration of these monocytes in response to hCPC and IFNγ-hCPC conditioned media. Both INCB 3284 dimesylate and anti-CCL2 decreased by at least twofolds the migration of CD14^+^CD16^−^ monocytes while IgG control did not have any significant effect (Figure 1D).

Together these results indicate that allogeneic hCPC at steady state or under inflammatory conditions have the competency to recruit circulating monocytes through the chemokine/receptor CCL-2/CCR2 axis.

### CD14^+^CD16^−^ Monocytes Develop an Anti-inflammatory Profile in the Presence of Allogeneic hCPC

**Figure 2:**
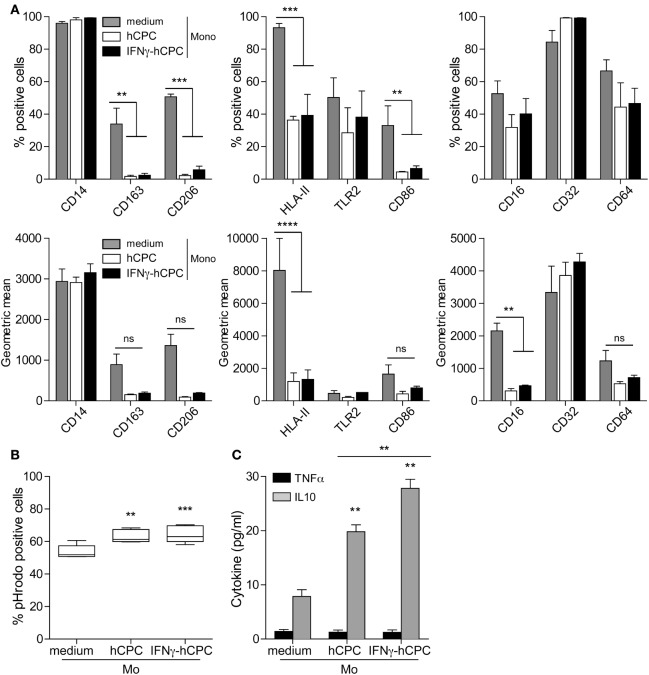
CD14^+^CD16^−^ monocytes develop an anti-inflammatory profile in the presence of allogeneic human cardiac-derived stem/progenitor cells (hCPC). CD14^+^CD16^−^ monocytes were cultured for 5 days in the absence (medium) or the presence of hCPC (hCPC) or interferonγ (IFNγ)-hCPC (IFNγ-hCPC) at a ratio of 5:1. **(A)** Expression of informative markers presented as percentage of positive cells (upper panel) and geometric mean (lower panel) was determined by flow cytometry. **(B)** Phagocytic activity was evaluated using Red pHrodo Zymozan A BioPaticles phagocytosis assay. Results are presented as percentage of pHrodo-positive cells. **(C)** TNFα and IL10 secretion by monocytes populations as determined by specific enzyme-linked immunosorbent assay. Data on the graphs represent the mean ± SEM from five independent experiments conducted with peripheral blood mononuclear cells isolated from five different donors against the same hCPC and are representative of data obtained with two other hCPC from different donors. Each data point represents the mean of experimental triplicates. *P < 0.05; **P < 0.01; ***P < 0.001; ****P < 0.0001; ns: not significant.

Having established that allogeneic hCPC could promote the recruitment of monocytes to the injured tissue, we next examined the phenotypic and functional changes that might occur under both steady state and inflammatory conditions. CD14^+^CD16^−^ monocytes were cultured for 5 days either alone or in the presence of hCPC or IFNγ-hCPC then phenotypically analyzed by flow cytometry. For a comprehensive assessment, we monitored both, the percentage of cells expressing a given marker and the intensity/quantity of the molecules displayed by positive and negative cells (geometric mean fluorescence intensity). The plastic adherence induced CD16, CD163, and CD206 expression in a considerable percentage of monocytes (Figure 2A; Figure S2 in Supplementary Material). Moreover, it increased the expression level of CD14, CD64, CD32, and HLA II and decreased that of TLR-2 and CD86 (Figure 2A; Figure S2 in Supplementary Material). In the presence of hCPC or IFNγ-hCPC, monocytes maintained bright expression of CD14 and CD32 but dampened the expression of CD16 and HLA II. They also showed significant lower expression of CD86 and TLR2 and neither expressed CD163 nor CD206 (Figure 2A; Figure S2 in Supplementary Material). The presence of hCPCs also endowed CD14^+^CD16^−^ monocytes with higher phagocytic activity, monitored by phagocytosis of pHrodo zymosan-A bioparticles (Figure 2B), and significant enhancement of IL10 secretion (Figure 2C).

Together these data suggest that hCPC at steady state or under inflammatory conditions could influence the effector functions of monocytes recruited to the site of injury.

### hCPC Impact CD14^+^CD16^−^ Monocytes Differentiation/Polarization Programs toward a Phenotype Overlapping with M2c Phenotype

**Figure 3:**
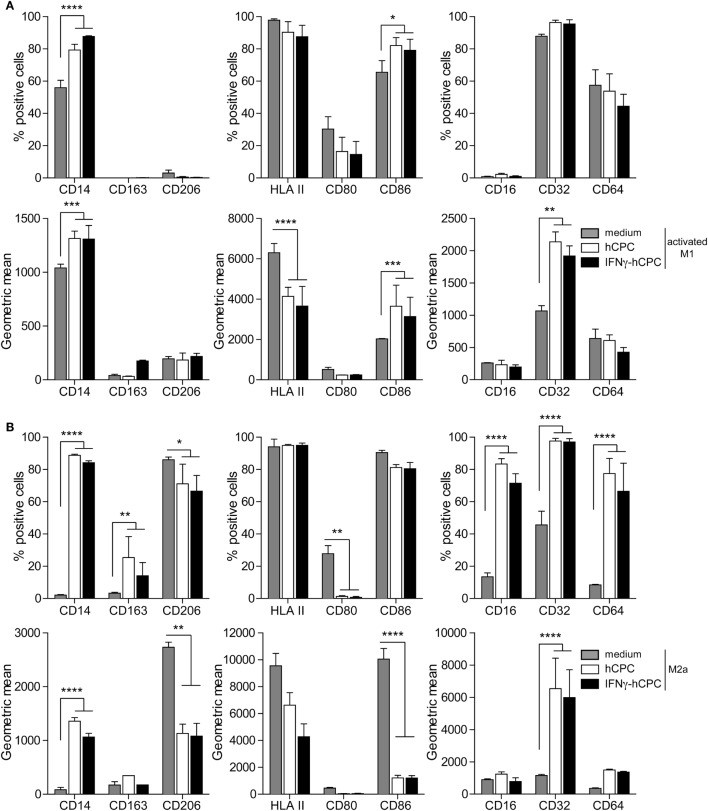
Allogeneic human cardiac-derived stem/progenitor cells (hCPC) impact CD14^+^CD16^−^ monocytes differentiation/polarization programs. CD14^+^CD16^−^ monocytes were differentiated/polarized with a cocktail of macrophage colony-stimulating factor (M-CSF)/interferonγ (IFNγ)/lipopolysaccharide (LPS) **(A)** or M-CSF/IL-4/IL-13 **(B)** in the absence (medium) or the presence of hCPC (hCPC) or IFNγ-hCPC (IFNγ-hCPC) for 5 days. Then by flow cytometry, we determined **(A)** expression of relevant markers of activated-M1 presented as percentage of positive cells (upper panel) and geometric mean (lower panel). **(B)** Expression of relevant markers of M2a presented as percentage of positive cells (upper panel) and geometric mean (lower panel). Data on the graphs represent the mean ± SEM from five independent experiments conducted with peripheral blood mononuclear cells (PBMC) isolated from five different donors against the same hCPC and are representative of data obtained with two other hCPC from different donors. Each data point represents the mean of experimental triplicates. *P < 0.05; **P < 0.01; ***P < 0.001; ****P < 0.0001; ns: not significant.

To understand whether allogeneic hCPC could influence the polarization/activation of monocytes, we performed co-culture experiments using M-CSF/IFNγ/LPS or M-CSF/IL4/IL13 cocktails in the absence or the presence of cell-to-cell contact settings with allogeneic hCPC or interferonγ (IFNγ)-hCPC. After 5 days of co-culturing, cells were analyzed by flow cytometry for the expression of informative markers. Under M-CSF/LPS/IFNγ conditions, monocytes displayed activated-M1 macrophage profile (thereafter activated-M1) (24) whereas M-CSF/IL4/IL13 promoted the polarization into M2a macrophage profile (thereafter M2a) (24) (Figure 3A). Substantial percentage of activated-M1 expressed high levels of CD14, CD86, HLA-II, and CD32, but was negative for CD16, CD163, and CD206. Low to fair percentage of cells also expressed CD80, CD64 albeit at low levels (Figure 3A upper panel and Figure S3A in Supplementary Material left panel). Most of M2a displayed moderate to high expression levels of CD86, HLA-II, and CD206. However, the M2a polarized cells did not display any significant expression of CD80, CD16, CD64, or CD163 but nearly 45% of them expressed low levels of CD32 (Figure 3A lower panel and Figure S3B in Supplementary Material right panel).

The presence of hCPC or IFNγ-hCPC impacted both CD14^+^CD16^−^ monocytes differentiation/polarization programs in a similar manner. Under the activated-M1 program (thereafter activated-M1^hCPC^), the presence of hCPC or IFNγ-hCPC increased the percentage and/or expression levels of CD14, CD86, and CD32, and dampened the HLA class II expression levels (Figure 3A upper panel and Figure S3B in Supplementary Material lower panel). Under M2a program (thereafter M2a^hCPC^), the presence of hCPC or IFNγ-hCPC induced increased percentage and/or expression level of CD14, CD32, CD64, and CD16, and dampened the CD86, HLA II, and CD206 expression levels (Figure 3A lower panel and Figure S3B in Supplementary Material right panel). Comparable effects were observed when the activation was conducted in the absence of cell-to-cell contact with hCPC using transwells (data not shown), albeit at less pronounced effects for both activated-M1^hCPC^ and -M2a^hCPC^.

In summary, under steady state or inflammatory conditions, hCPC fine-tune the activation/polarization of CD14^+^CD16^−^ monocytes toward a phenotype marked by high expression levels of CD14 and the Fcγ receptors CD32 and CD64, lower levels of the mannose receptor CD206 and immune relevant molecules CD80, CD86 and HLA class II, and absence of the scavenger receptor CD163; a profile overlapping with M2c macrophages phenotype (24).

### hCPC Fine-Tune CD14^+^CD16^−^ Monocyte-Derived M1 and M2a Macrophages Functioning toward Anti-inflammatory/Immune-Regulatory Macrophages

We then investigated whether the presence of hCPC would also fine-tune the functioning of CD14^+^CD16^−^ monocyte-derived activated-M1 and Ma2 as it did for their phenotype.

**Figure 4:**
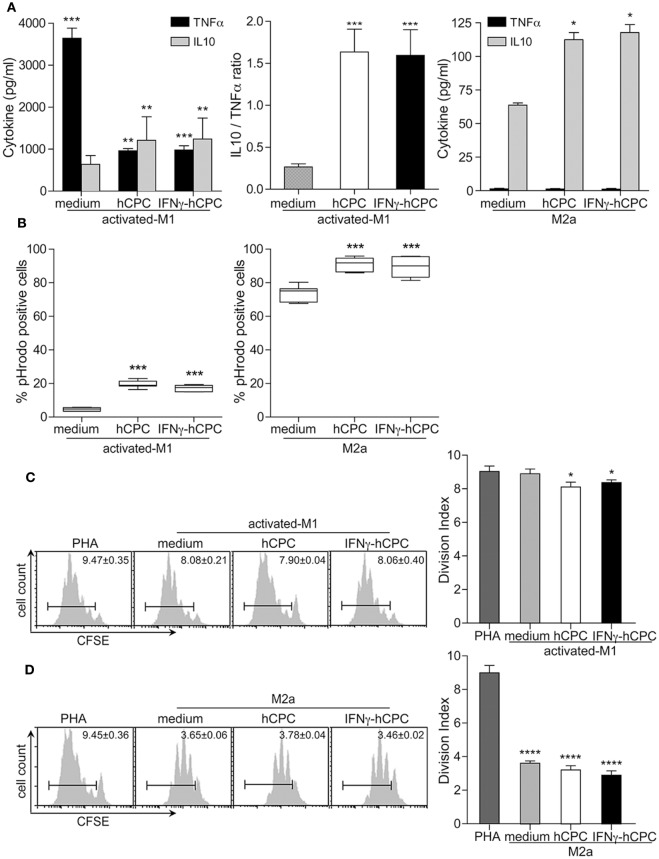
Human cardiac-derived stem/progenitor cells (hCPC) promote IL10 production, influence phagocytic activity, and previse immuno-regulatory capacity of CD14^+^CD16^–^ derived macrophages. CD14^+^CD16^−^ monocytes were differentiated/polarized/activated as in Figure 3 in the presence **(A,B)** or absence **(C,D)** of cell-cell contact. **(A)** TNFα and IL10 secretion by CD14^+^CD16^−^ monocyte-derived activated-M1 (left panel) and CD14^+^CD16^−^ monocyte-derived M2a (right panel) in the presence or absence of hCPC, as evaluated by specific enzyme-linked immunosorbent assay. **(B)** Phagocytic activity of CD14^+^CD16^−^ monocyte-derived activated-M1 (left panel) and CD14^+^CD16^−^ monocyte-derived M2a (right panel) in the presence or absence of hCPC was determined by Red pHrodo Zymozan A BioPaticles phagocytosis assay. hCPC or interferonγ (IFNγ)-hCPC were compared to medium. **(C)** Modulation of PHA-induced T cells proliferation by activated-M1 or **(D)** M2a in the absence or the presence of hCPC was determined by tailored immunomodulation assays as described under Section “Materials and Methods.” T cells proliferation under different experimental conditions was evaluated by measuring the loss of carboxyfluorescein succinimidyl ester (CFSE) intensity on 7-ADD-negative CFSE-labeled CD3^+^ T cells using flow cytometry. Results are presented as division index compared to PHA (right panels) and representative histograms in the left panels. Data on the graphs represent the mean ± SEM from five independent experiments conducted with peripheral blood mononuclear cells (PBMC) isolated from five different donors against the same hCPC and are representative of data obtained with two other hCPC from different donors. Each data point represents the mean of experimental triplicates. *P < 0.05; **P < 0.01; ***P < 0.001; ****P < 0.0001; ns: not significant.

M2c macrophages are potent producers of the anti-inflammatory cytokine IL10 involved in immune-regulation, matrix deposition and tissue remodeling (18, 24). Accordingly, we assessed the IL10 production likened to pro-inflammatory cytokine TNFα by activated-M1 and M2a compared to activated-M1^hCPC^ and M2a^hCPC^. Under activating/polarizing conditions, activated-M1 produced high amounts of TNFα and very low amounts of IL10, whereas M2a mainly produced IL10 and no TNFα (Figure 4A). Activated-M1^hCPC^ showed significant decreases in TNFα production and enhanced secretion of IL10 (Figure 4A left and middle panels). Similar to M2a, M2a^hCPC^ secreted mainly IL10 (Figure 4A right panel). Comparable patterns were observed when co-cultures were performed in transwell settings (data not shown). Neither hCPC nor IFNγ-hCPC produce IL10 (11) and their culture under the monocytes activation/polarization conditions neither induced the production of IL10 nor TNFα (data not shown).

We then assessed the impact of hCPC on phagocytosis and modulation of immune inflammatory response, two effector functions of classically activated and alternatively activated macrophages.

CD14^+^CD16^−^ monocytes were activated as above toward activated-M1 and -M2a macrophages in the presence or absence of hCPC or IFNγ-hCPC for 5 days, then their phagocytic activity and capacity to modulate an ongoing adaptive immune response were examined. In accordance with their scavenging properties (Figure 4B), activated-M1 showed particularly weak phagocytic activity compared to M2a, around 4.5 and 80% pHrodo-positive cells, respectively (Figure 4B left panel). Yet, the presence of hCPC or IFNγ-hCPC yielded an approximate fourfold increase in activated-M1 phagocytic activity, nearly 20% of activated-M1^hCPC^ were positive for pHrodo (Figure 4B left panel). Although M2a are prevailing phagocytes, M2a^hCPC^ were endowed with higher phagocytic activity as evidenced by significantly higher percentage of pHrodo-positive M2a^hCPC^ cells compared to M2a (Figure 4B right panel). Thus, at steady state or under inflammatory conditions hCPC-driven reshaping of monocytes/macrophages response results in enhanced phagocytic activity.

Next, we investigated the impact of CD14^+^CD16^−^ monocyte-derived macrophage populations on an ongoing T cell response. CFSE-labeled autologous PBL were stimulated with PHA, polyclonal T cell activator, in the presence or absence of activated-M1 or activated-M1^hCPC^ (steady state and inflammatory). Co-cultures were carried out in transwell settings for 5 days and T cell proliferation was monitored by flow cytometry. The presence of activated-M1 had no effect on PHA-induced T cell proliferation. Similarly, the effect of activated-M1^hCPC^ on PHA-induced T cells proliferation was also marginal (Figure 4C). By contrast, M2a macrophages demonstrated high capacity to downregulate PHA-induced T cells proliferation. M2a^hCPC^ similarly downregulated T cells proliferation and did not demonstrated significant advantage over M2a macrophages (Figure 4D). Accordingly, hCPC do not promote but maintain the anti-inflammatory/immune-modulatory capacity of macrophages.

Together these results indicate that hCPC, at steady state or under inflammatory conditions, fine-tune the differentiation/polarization of CD14^+^CD16^−^ monocytes toward anti-inflammatory/immune-regulatory macrophages marked by enhanced production of IL10.

### hCPC Bend CD14^+^CD16^−^ Monocytes Differentiation to Immature DC toward Macrophage-Like Features

Recent evidence pointed to the pivotal role that DC could have in remodeling and cardiac function after MI and pinpointed tolerogenic DC as important initiators of the inflammatory-to-reparative response (19, 20, 23). Therefore, we checked the impact of hCPC on the behavior of CD14^+^CD16^–^ derived DC.

**Figure 5:**
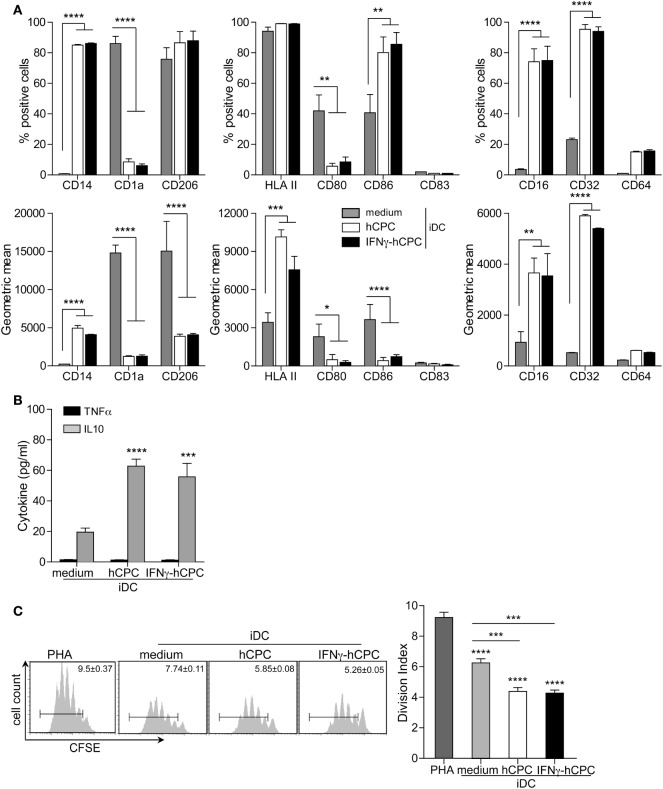
CD14^+^CD16^−^ monocytes-derived immature cells (iDC) acquire a macrophage-like profile in the presence of allogeneic human cardiac-derived stem/progenitor cells (hCPC). CD14^+^CD16^−^ monocytes were differentiated into iDC with a GM-CSF/IL-4 cocktail in the absence (medium) or presence of hCPC (hCPC) or interferonγ (IFNγ)-hCPC (IFNγ-hCPC) at a ratio of 5:1. Co-cultures were conducted in the presence **(A,B)** or absence of cell–cell contact **(C)**. **(A)** Expression of relevant iDC markers (left panel), of dendritic cell maturation markers (middle panel), and of FCγR (right panel) as determined by flow cytometry. Results are presented as the percentage of positive cells (upper panel) and geometric mean (lower panel). **(B)** IL10 and TNFα secretion by CD14^+^CD16^−^ monocyte-derived iDC in the absence (medium) or the presence of hCPC (hCPC) or IFNγ-hCPC (IFNγ-hCPC) determined by specific enzyme-linked immunosorbent assay. **(C)** Modulation of PHA-induced T cells proliferation by CD14^+^CD16^−^ monocyte-derived iDC in the absence (medium) or the presence of hCPC (hCPC) or IFNγ-hCPC (IFNγ-hCPC). T cells proliferation was analyzed by flow cytometry as described in Figure 4. Results are presented as division index compared to PHA or medium (right panel) and representative histograms in the left panel. Data on the graphs represent the mean ± SEM from five independent experiments conducted with PBMC isolated from five different donors against the same hCPC and are representative of data obtained with two other hCPC from different donors. Each data point represents the mean of experimental triplicates. *P < 0.05; **P < 0.01; ***P < 0.001; ****P < 0.0001.

The presence of GM-CSF/IL4 cocktail induces the differentiation of CD14^+^CD16^−^ monocytes into DC expressing relevant markers of immature cells (iDC); negative for both CD14 and the DC maturation marker CD83 (Figure 5A). These differentiated cells are bright for CD1a and CD206 and they express fair levels of HLA II, CD80, CD86 (Figure 5A; Figure S4 in Supplementary Material). iDC generated in the presence of hCPC or IFNγ-hCPC (thereafter iDC^hCPC^) displayed significantly altered phenotype. Compared to iDC, iDC^hCPC^ displayed significant expression of CD14 (*p* < 0.0001), brighter expression of HLA II (*p* < 0.0001), significant bright expression of FCγR CD16 and CD32 (*p* < 0.01 and 0.001, respectively), significantly dimmer expression of CD80 and CD86, and maintained considerable level of CD206 (Figure 5A; Figure S4B in Supplementary Material right panel). This phenotypic change was accompanied by significantly increased production of IL10; iDC^hCPC^ secreted threefolds more IL10 than iDC while both displayed negligible secretion of TNFα (Figure 5B).

Since iDC do not foster high immune-stimulatory capacity, we next checked whether our CD14^+^CD16^−^ monocyte-derived iDC could interfere with T cell proliferation. Indeed, iDC failed to activate allogeneic T cells (Figure S5 in Supplementary Material) but significantly reduced the proliferation of autologous PHA-induced T cells (Figure 5C). Similarly, iDC^hCPC^ failed to activate allogeneic T cells (Figure S5A in Supplementary Material) but were more potent than iDC in reducing the proliferation of autologous PHA-induced T cells (Figure 5C).

Together these results indicate that differentiation of CD14^+^CD16^−^ monocytes to iDC in the presence of hCPC, at steady state or under inflammatory conditions, shifted iDC differentiation toward a macrophage-like phenotype with anti-inflammatory/immune-modulatory features.

### Mature DC^hCPC^ Are Immune-Modulators Rather Than T Cells Activators

**Figure 6:**
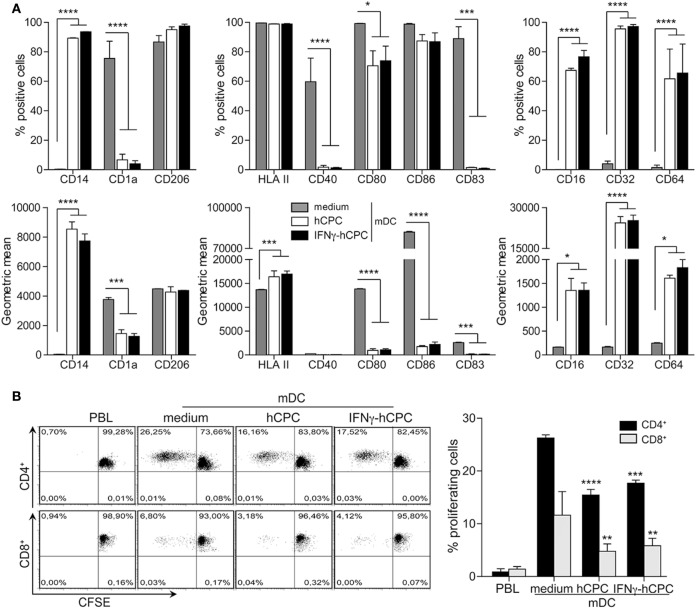
Human cardiac-derived stem/progenitor cells (hCPC) confer macrophage-like profile and impair the capacity of mature DC (mDC) to induce allogeneic T cells proliferation. CD14^+^CD16^−^ monocytes were differentiated into immature cells as in Figure 5 then their maturation to mDC was induced with lipopolysaccharide (LPS). **(A)** Expression of relevant markers (left panel), of dendritic cell maturation markers (middle panel), and of FCγR (right panel) by CD14^+^CD16^−^ monocyte-derived mDC in the absence (medium) or the presence of hCPC (hCPC) or IFNγ-hCPC (IFNγ-hCPC) as determined by flow cytometry. Results are presented as the percentage of positive cells (upper panel) and geometric mean (lower panel). **(B)** CD14^+^CD16^−^ monocyte-derived mDC in the absence (medium) or the presence of hCPC (hCPC) or IFNγ-hCPC (IFNγ-hCPC) were used to stimulate the proliferation of allogeneic carboxyfluorescein succinimidyl ester (CFSE)-labeled peripheral blood lymphocyte (PBL) at a PBL/DC ratio of 5:1 as described under Section “Materials and Methods.” The percentage of CD4^+^ and CD8^+^ proliferating T-cells was determined by flow cytometry as described in Figure 4. Results are presented as representative dot plots of proliferating cells (left panel). Histograms (right panel) represent % of proliferating cells compared to medium. Data on the graphs represent the mean ± SEM from four independent experiments conducted with peripheral blood mononuclear cell (PBMC) isolated from four different donors against the same hCPC and are representative of data obtained with two other hCPC from different donors. Each data point represents the mean of experimental triplicates. **P < 0.01; ***P < 0.001; ****P < 0.0001.

We then asked whether hCPC could device the behavior of mDC, potent immune activators of adaptive T cells response. We differentiated CD14^+^CD16^−^ monocytes to iDC in the presence or absence of hCPC then activated them with LPS. mDC displayed classical phenotype with expression of the maturation marker CD83, bright expression of HLA II and co-stimulatory CD80 and CD86 molecules, and expression of CD40 and maintained CD1a and CD206 (Figure 6A). hCPC at steady state or under inflammatory conditions compromised the maturation of DC in favor of macrophage lineage, evidenced by considerable expression of CD14, drastic loss of CD1a and CD83, by nearly all mDC^hCPC^ and expression of FCγ receptors by a considerable percentage of cells (Figure 6A).

As chief activators of T cells, mDC significantly activated allogeneic CD4^+^ and to a lesser extent CD8^+^ (Figure 6B; Figure S6A in Supplementary Material). Compared to mDC, mDC^hCPC^ demonstrated significant reduced capacity to activate allogeneic T cells; the proliferation of both CD4^+^ and CD8^+^ allogeneic T cells in response to mDC^hCPC^ was nearly 1.6-fold less than to mDC (Figure 6B right panel and Figure S6A in Supplementary Material).

**Figure 7:**
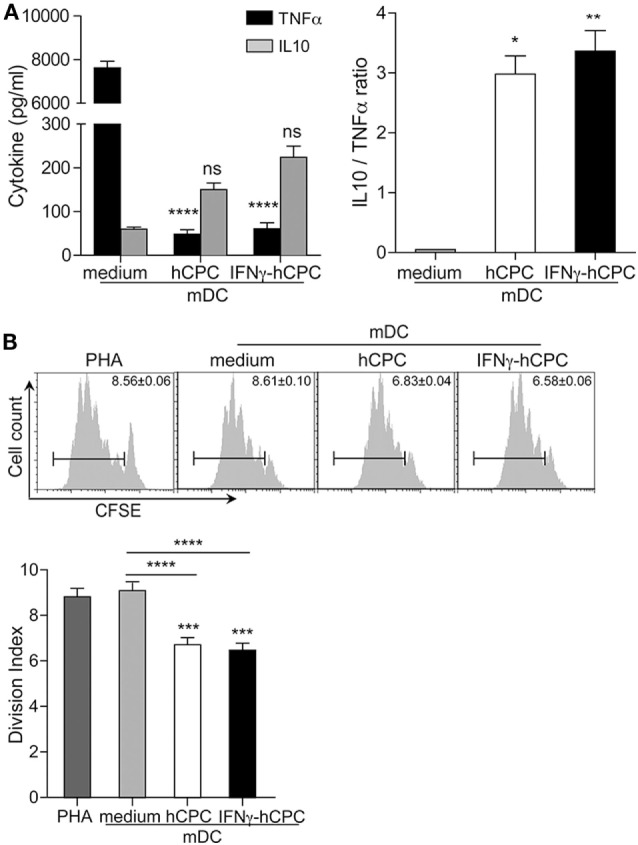
mDC^hCPC^ and mDC^IFNγ-hCPC^ produce IL10 rather than TNFα and immune-modulate an ongoing immune response. **(A)** The levels of IL10 and TNFα were quantified by specific enzyme-linked immunosorbent assay from supernatants of co-cultures conducted as in Figure 6. **(B)** Modulation of PHA-induced T cells proliferation by mature DC (mDC) in the absence (medium) or the presence of hCPC (hCPC) or IFNγ-hCPC (IFNγ-hCPC) was determined by immunomodulation assays described under Section “Materials and Methods.” T cells proliferation was analyzed by flow cytometry as in Figure 4. Results are presented as division index compared to PHA or medium (lower panel) and representative histograms in the upper panel. Data on the graphs represent the mean ± SEM from four independent experiments conducted with Peripheral blood mononuclear cells (PBMC) isolated from four different donors and are representative of data obtained with two other hCPC from different donors. Each data point represents the mean of experimental triplicates. *P < 0.05; **P < 0.01; ***P < 0.001; ****P < 0.0001; ns: not significant.

Accordingly, we tested whether mDC^hCPC^ have acquired immune-modulatory capacity. We measured the production of TNFα likened to IL10 by mDC and mDC^hCPC^. As established, mDC produced mainly TNFα and modest amount of IL10 (Figure 7A). In comparison, mDC^hCPC^ produced higher amounts of IL10 and very modest amount of TNFα (Figure 7A). Thus, the presence of hCPC during maturation of DC biases their production of inflammatory TNFα in favor of IL10, which might endow them with some immune-modulatory capacity. Indeed, while mDC did not down regulate the PHA-induced T cells proliferation, mDC^hCPC^ reduced their proliferation by nearly 30% (Figure 7B).

Thus, the presence of hCPC during maturation of DC biases their phenotype and impairs their immune activator function driving the generation of cells with anti-inflammatory/immune-regulatory macrophage-like features.

## Discussion

In addition to off-the-shelf availability, allogeneic hCPC-based therapy are considered a potential therapeutic alternative for cardiac repair likely by shaping the local immune response in a constructive paracrine manner (13). In order to deepen these mechanisms, we investigated here the potential impact of allogeneic hCPC on CD14^+^CD16^−^ monocytes, crucial cells for the regulation of the inflammation and the healing process. Collectively, we demonstrate that allogeneic hCPC reshape the macrophages and DCs activities by favoring anti-inflammatory/tolerogenic differentiation and activation/polarization of their parental CD14^+^CD16^−^ monocytes, further suggesting that therapeutic allogeneic hCPC might contribute to post-infarct myocardial healing *via* their regulation of M1/M2 macrophages and immature/mature DCs activities.

Our results demonstrated that allogeneic hCPC at steady state or within inflammatory environment have the capacity to recruit CD14^+^CD16^−^ monocytes *via* CCL-2/CCR2 pathway. The development and functions of these lineage-committed cells are regulated by growth factors and interleukins. Their recruitment is mandatory for tissue healing, but if uncontrolled, these cells might exacerbate inflammation and compromise reparation. In this context, many strategies were proposed to control their recruitment without disturbing physiological inflammation resolution (30–32). The role of monocytes in MI and post-MI healing is a double-edged sword. Within this notion, it is conceivable that the capacity of allogeneic hCPC to produce CCL-2 and to recruit CD14^+^CD16^−^ monocytes is important to assist the immune cells recruited to the injured myocardium in creating an inflammatory microenvironment that is favorable for cardiac repair/regeneration but with minimal interferences to their inflammatory activities at a specific time point. Indeed, we also found that upon culture with hCPC, monocytes acquire a dominant reparative behavior at the expense of the inflammatory one, as evidenced by the higher phagocytic activity and production of anti-inflammatory IL10. CCL-2 is essential not only for monocytes recruitment but also for angiogenesis, arteriogenesis, collagen expression, and regulation of matrix metalloproteinases (33–35). Thus, allogeneic hCPC harness the inflammatory events by inciting a “controlled recruitment of monocytes,” which would enhance rather than disturb healing and repair.

The presence of hCPC impaired also the commitment and differentiation pathways of monocytes. Independently of M1 or M2a differentiation program, our results showed that the allogeneic hCPC instruct macrophages to adopt anti-inflammatory pro-resolving and tissue-regenerating/repairing properties. M1^hCPC^ and M2a^hCPC^ displayed a phenotype comparable more or less to that of M2c tissue-repairing macrophages (24), restrained inflammatory TNFα production and increased significantly IL10 secretion and phagocytic activity. We did not investigate the mechanisms through which this fine-tuning might occur. However, in the absence of cell–cell contact the effects and patterns were only less pronounced than those occurring within cell–cell contact settings. Although warranted further investigations it is likely that hCPC-driven monocyte polarization implicates paracrine mechanisms that are probably reinforced by juxtacrine signaling occurring during cell–cell contact.

Interleukin 10 is a key anti-inflammatory cytokine with recognized effects on immune and non-immune cells and has been implicated in the regulation of post-infarction inflammatory response (36–38). The rapid conversion of pro-inflammatory TNFα-producing macrophages to an anti-inflammatory IL10-producing phenotype appears to be critical for long-term survival of stem/progenitor cell population in most tissues. Allogeneic hCPC affect monocytes polarization to TNFα-producing activated-M1 macrophages converting them to IL10-producing phenotype. This might contribute to maintaining long-term survival of endogenous stem/progenitor pool in the myocardium. However, it does not appear effective in down regulating an ongoing adaptive immune response and at the most might contribute to maintaining proper functioning of immune-modulator cells such as M2 macrophages. Thus, infusion of allogeneic hCPC might reshape macrophage response within injured myocardium by favoring M2c-like polarization that might result in increased capacity of M2 tissue-repairing macrophages, which would promote the reparative/regenerative process.

Phagocytosis is defined as the process of recognition and engulfment of microorganisms, dead cells, and cellular debris that accumulate during infection and injury. It plays a critical role in the maintenance of homeostasis during fundamental biological processes such as normal tissue turnover, remodeling of tissues development of the immune system and resolution of inflammation (39). The recognition of ligands expressed on the target by specific receptors on the ingesting cells. The elimination of apoptotic cells, debris, and others pro-inflammatory mediators by phagocyte cells stop the infiltration by immune cells and prevent the release of the cellular contents in the milieu by dead cells and clear the microenvironment; thus, contribute to the resolution of inflammation and reestablish tissue homeostasis. Hence, allogeneic hCPC promote inflammation resolution by tuning activation/polarization toward macrophages endowed with pronounced phagocytic activity. However, activated-M1^hCPC^ did not express classical phagocyte receptors CD163 or CD206. M2a^hCPC^ macrophages only expressed dim CD206 similar to M2c macrophages, which are recognized as key anti-inflammatory, immune-modulatory, and tissue-repairing macrophages. Differences in expression levels of these receptors by macrophage subsets in different culturing conditions were previously reported (24). The absence of these two receptors in our hands is, therefore, probably related to our experimental conditions. Nonetheless, the ability of macrophages to mediate phagocytosis seems to relay more heavily on their activation status rather than the expression level of specific receptors (40). Therefore, the higher phagocytic activity of activated-M1^hCPC^ and M2a^hCPC^ compared to activated-M1 and -M2a reported herein might be linked to their higher expression of Fcγ receptors in particular FcγRII CD32 that might be indicative of a particular activation state empowered by higher phagocytosis rather than mediating phagocytosis.

Similarly to macrophage differentiation experiments, hCPC exerted a potent inhibition on CD14^+^CD16^−^ monocytes differentiation to CD1a^+^ DC and on their maturation in the presence of LPS. iDC^hCPC^ and mDC^hCPC^ acquired a more or less M2-like macrophage phenotype in the presence of hCPC, expressing higher levels of Fcγ receptors, mannose receptor CD206 and CD14, but considerably lower levels of co-stimulatory CD80, and CD86 as well as antigen-presenting elements HLA class II molecules. Beyond this impaired phenotype, mDC^hCPC^ have almost lost the capacity to activate allogeneic CD4^+^ or CD8^+^ T cells. However, our results also showed that both iDC^hCPC^ and mDC^hCPC^ have the capacity to downregulate ongoing activation/proliferation of autologous T-cell and produce mainly IL10. This suggests that infusion of allogeneic hCPC might, either directly (11) or indirectly, regulate the activity of inflammatory T cells infiltrating the injured myocardium through their effect on the differentiation of DC.

Emerging evidence indicates the importance of DC in both post-MI inflammation and the subsequent healing process. The correlation between the degree of DC infiltration and the extent of post-infarction healing process following human heart infarction suggested a protective role of DC post-MI inflammation (20). In a murine experimental model, it has been shown that mDC from infarcted heart have activated phenotype and are loaded with self-antigen licensing them for efficient auto-reactive T cell activation (41). Indeed, dangerous signals released by tissue injury can activate DC and generate adaptive response. Although not yet fully examined in humans, potential MI therapeutic approaches should, therefore, ideally envisage keeping these DCs in check preventing their maturation to limit post-MI immune-mediated tissue destruction. Furthermore, treatment of MI mice with tolerogenic DC limited post-infarct left ventricular remodeling likely by inducing a systemic activation of regulatory T cells and eliciting an inflammatory-to-reparative macrophage shift (23). In this context, allogeneic hCPC might appear as interesting approach since they can (1) impair monocytes differentiation to iDC, (2) prevent iDC maturation, and (3) impair mDC proper functioning biasing it toward regulatory “tolerogenic DC” functioning, thus hCPC could elicit an inflammatory-to-reparative macrophage shift.

The role of myeloid cells in allogeneic recognition and allogeneic transplantations has been minimally studied (42). Nonetheless, mouse monocytes mount a greater inflammatory response to allogeneic cells than to syngeneic cells (43), suggesting that monocytes are able to distinguish self from non-self tissues. In murine models of allogeneic grafts, the allogeneic non-self recognition by monocytes also elicited persistence differentiation of monocytes into mDCs and stimulated allogeneic adaptive response (44). These findings underscored the implication of non-self by the monocytes in initiating allogeneic reactions and the ultimate outcome of graft rejection. Within these findings, it is conceivable that monocytes in our model also distinguished allogeneic hCPC as non-self eliciting their differentiation to anti-inflammatory “tolerogenic” innate myeloid cells that stimulated modulatory anti-inflammatory adaptive response. In perfect adequacy, our previous findings demonstrating that allogeneic recognition of hCPC by adaptive immune cells elicits a non-classical allogeneic regulatory response rather than classical inflammatory allogeneic response (11, 12). Whether the degree of allogenicity might influence the hCPC-elicited responses is not yet clear. Nonetheless, within inflammatory states, cells are often more immunogenic given their higher expression of immune relevant molecules, in particular the HLA molecules. The capacity of IFNγ-primed hCPC expressing both HLA class I and II molecules and mimicking those under inflammatory conditions with higher immunogeneicity/allogenicity, to fine-tune the functioning of monocytes and their descendants was comparable to that of steady state hCPC albeit for enhanced capacity in shifting the balance of cytokine production toward anti-inflammatory IL10. This might imply a role for the degree of allogenicity in dictating the impact of hCPC on these innate cells but further investigations and a larger cohort are warranted.

In summary, our study demonstrates that allogeneic cardiac-derived stem/progenitor cells fine-tunes the differentiation and activation/polarization of key elements of the mononuclear phagocyte system, including circulating monocytes, macrophages, and DCs, toward anti-inflammatory immune-modulating behavior. Thereby, these induced modifications might contribute to the beneficial effects of the therapeutic allogeneic hCPC and could be employed in regenerative/reparative therapies.

## Ethics Statement

The study was approved by the local research ethical committee and “Comité consultatif pour la protection des personnes dans les recherches biomédicales.” All experiments were performed in accordance with the local institutional guidelines and regulations and the approval of the local ethic committee, and with informed consent from all subjects. Human cardiac biopsies were obtained from patients undergoing open-chest surgery after signed informed consent in accordance with the Declaration of Helsinki. The ethical committees of “Hospital 12 de Octubre” and “Fundación Jiménez Díaz” (Madrid), Spain have approved the project. Blood donors signed an informed consent following human ethics committee from the “Centro de transfusion de la Comunidad de Madrid” and the institution has approved the study.

## Author Contributions

ND, HH, and RM contributed to devising the study, designing the experiments, results interpretation, and to data analysis. DC, AB, IP, OD, and WD provided critical feedback. HEC and NJ-F gave critical feedback and revised the manuscript. EL and RA-D conceived, designed, supervised the work, and analyzed the data, revised, and approved the manuscript. All authors read and approved the final manuscript.

## Conflict of Interest Statement

ND, IP, OD, RM, WD, and EL are full-time employees of TiGenix. DC, AB, HEC, NJ-F, and RA-D declare no competing financial interests.
