# Supplementary material for: Human Cardiac-Derived Stem/Progenitor Cells Fine-Tune Monocyte-Derived Descendants Activities toward Cardiac Repair
Source: Front Immunol. 2017 Oct 26;8:1413. doi: 10.3389/fimmu.2017.01413 (PMC5662627; doi:10.3389/fimmu.2017.01413)
Supplement: Supplementary file 1 [file Data_Sheet_1.pdf]

Supplementary Figures and Tables

Human Cardiac-Derived Stem/Progenitor Cells Fine-Tune Monocyte-Derived Descendants Activities Toward Cardiac Repair

Noémie Dam[1,3], Hocine Rachid Hocine[3], Itziar Palacios[1], Olga delaRosa[2], Ramón Menta[1], Dominique Charron[3,4], Armand Bensussan[3], Hicham El Costa[5], Nabila Jabrane-Ferrat[5], Wilfried Dalemans[6], Eleuterio Lombardo[2*], Reem Al-Daccak[3,4*]

Supplementary Figure 1: ***$CD14^+CD16^-$ monocytes migrate in response to CCL-2 and hCPC-conditioned medium***

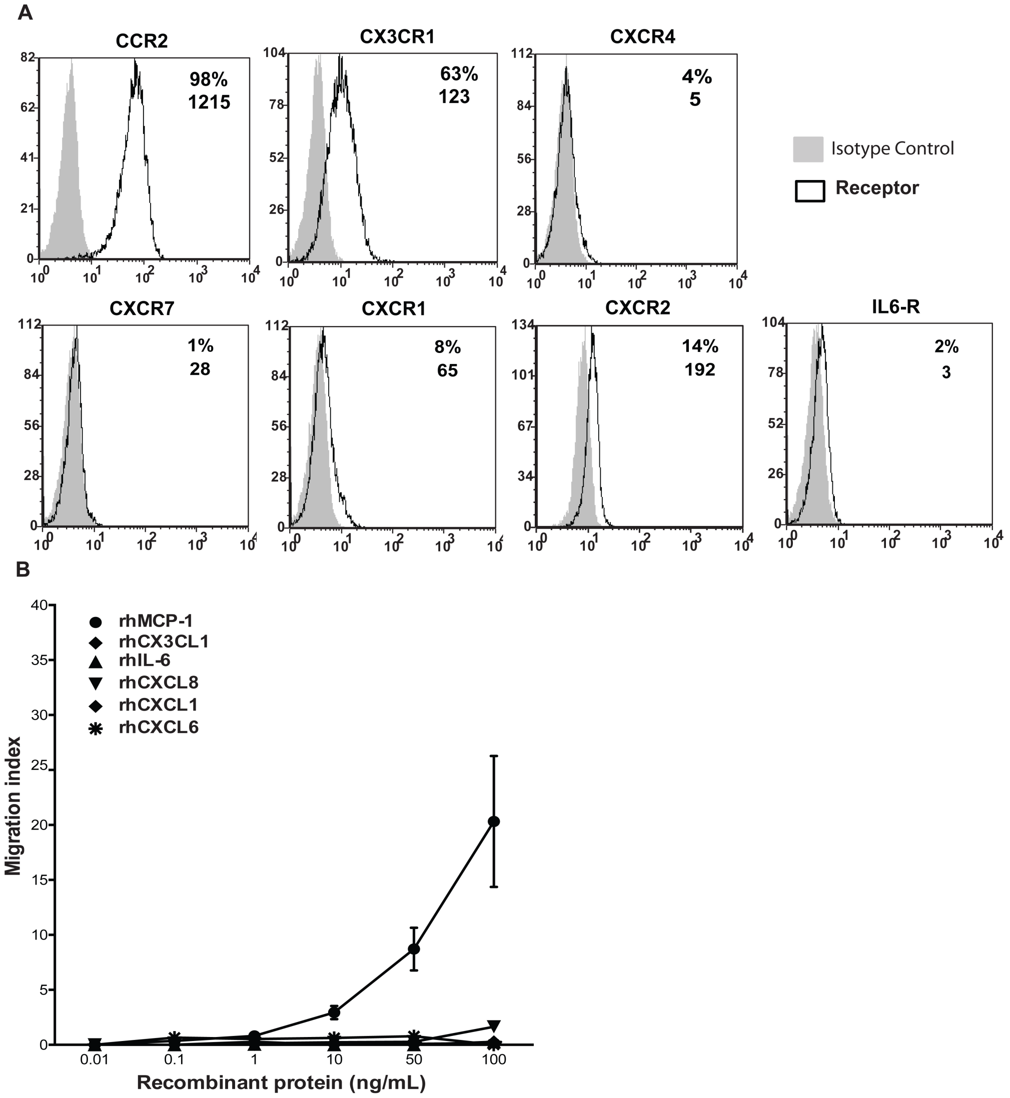

A) $CD14^+CD16^-$ monocytes were isolated from blood samples and their expression of various chemokine receptors was determined using specific antibodies and flow cytometry analysis. B) $CD14^+CD16^-$ monocytes migration in response to various recombinant chemokines was determined by flow cytometry in Boyden chambers with 5μm pores. Freshly isolated $CD14^+CD16^-$ monocytes were plated in the upper compartment whereas stimuli were loaded in the lower compartment of the transwell chambers. The number of migrated monocytes (into the lower compartment) was counted by flow cytometry and used to calculate the migration index as follow: number of cells migrated in presence of a stimulus/number of cells migrated in absence of stimulus.

Supplementary Figure 2: ***hCPC tend adherence-activated CD14^{+}CD16^{-} monocytes towards anti-inflammatory profile***

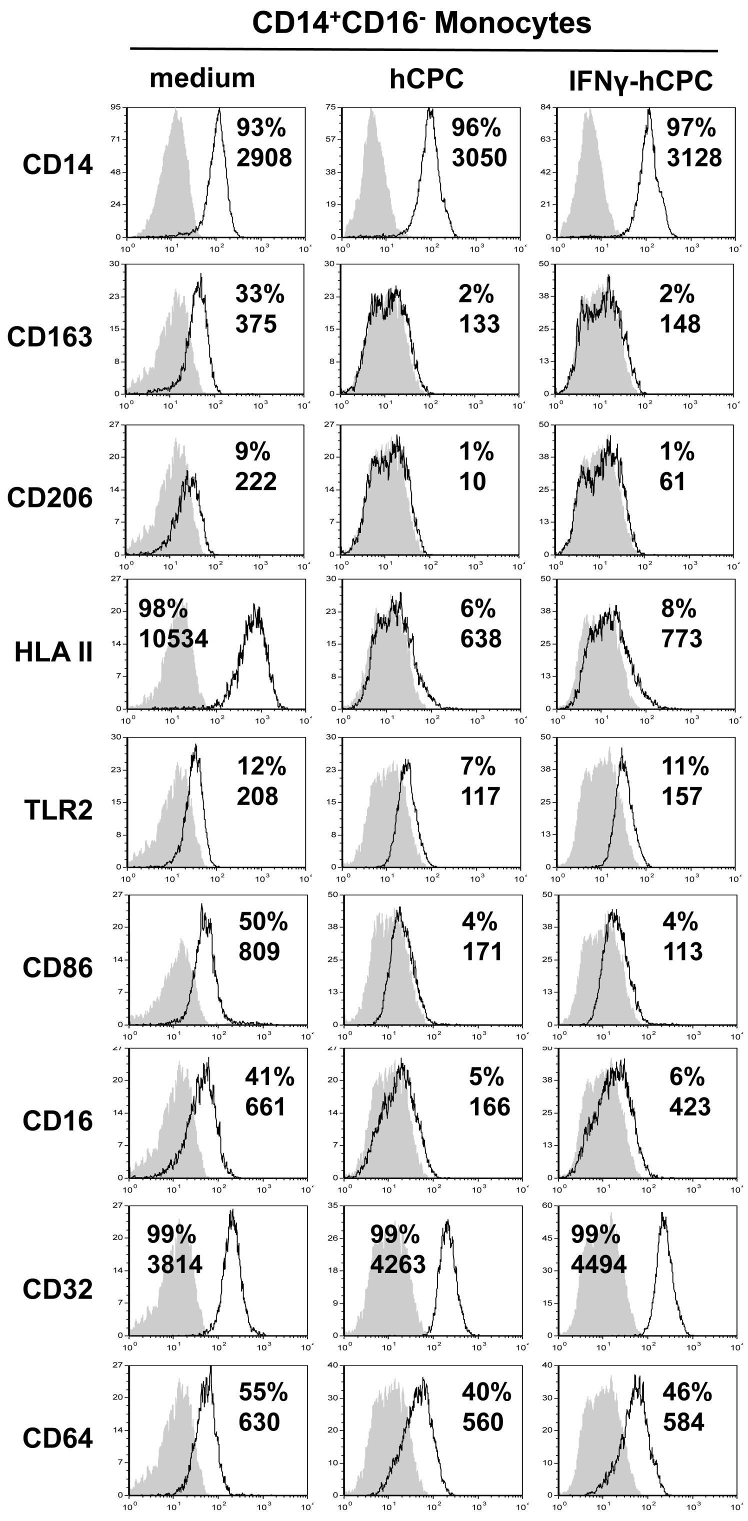

CD14^{+}CD16^{-} monocytes were cultured for 5 days in the absence (medium) or the presence of hCPC (hCPC) or IFNγ-hCPC (IFNγ-hCPC) at a ratio of 5:1 then the expression of informative markers was determined using specific antibodies and flow cytometry analysis. Representative histograms indicating the percentage of positive cells and geometric mean.

Supplementary Figure 3: ***Allogeneic hCPC fine-tune $CD14^{+}CD16^{-}$ monocytes polarization/activation***

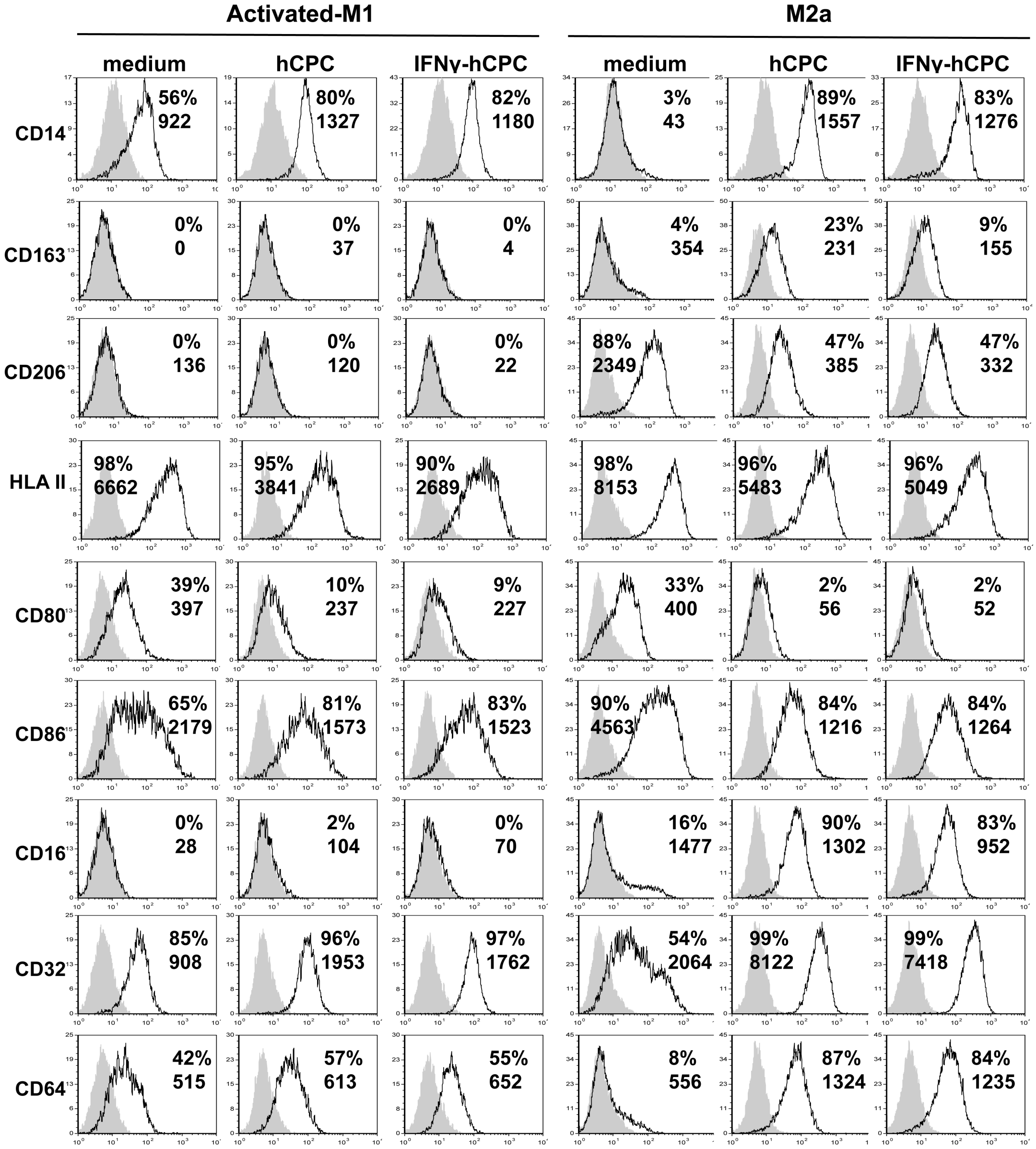

$CD14^{+}CD16^{-}$ monocytes were polarized/activated with a cocktail of M-CSF/IFNγ/LPS towards activated-M1 phenotype in the absence (medium) or the presence of hCPC (hCPC) or IFNγ-hCPC (IFNγ-hCPC) or with a cocktail of M-CSF/IL-4/IL-13 towards M2a phenotype in the absence (medium) or the presence of hCPC (hCPC) or IFNγ-hCPC (IFNγ-hCPC) for 5 days. The expression of informative markers was then determined using specific antibodies and flow cytometry analysis. Representative histograms indicating the percentage of positive cells and the geometric mean.

Supplementary Figure 4: ***CD14⁺CD16⁻ monocytes-derived DC acquire macrophage-like profile in the presence of allogeneic hCPC***

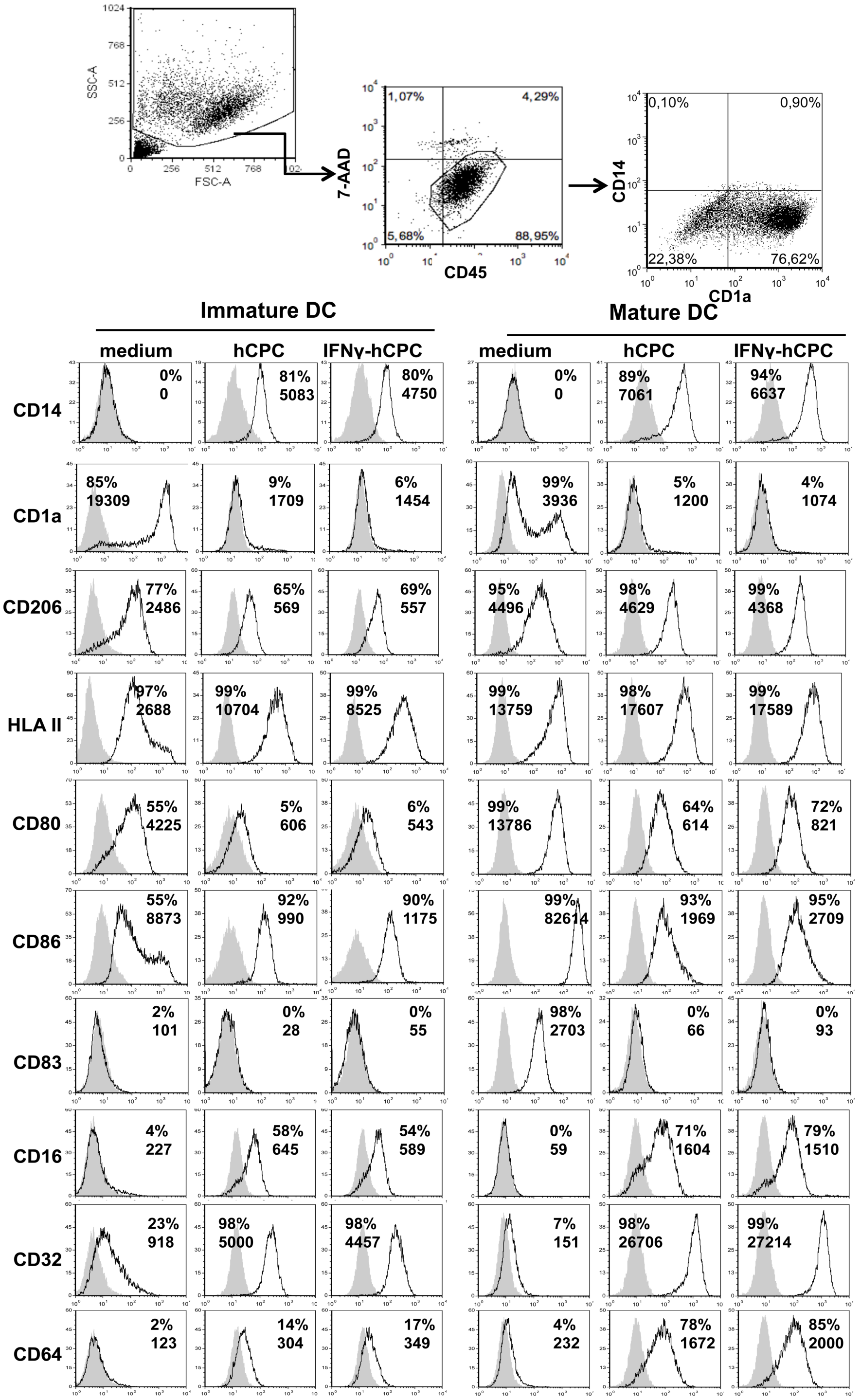

CD14⁺CD16⁻ monocytes were differentiated into DC with a GM-CSF/IL-4 cocktail (immature DC) in the absence (medium) or presence of hCPC (hCPC) or IFNγ-hCPC (IFNγ-hCPC) for 5 days at a ratio of 5:1. LPS was then added for another 2 days to induce the maturation of DC (mature DC) in the absence (medium) or the presence of hCPC (hCPC), or IFNγ-hCPC (IFNγ-hCPC). The expression of informative markers was then determined using specific antibodies and flow cytometry analysis. Representative histograms indicating the percentage of positive cells and the geometric mean.

Supplementary Figure 5: ***iDC, iDC^{hCPC}, and iDC$^{IFN\gamma\text{-}hCPC}$ are unable to stimulate allogeneic T cells proliferation***

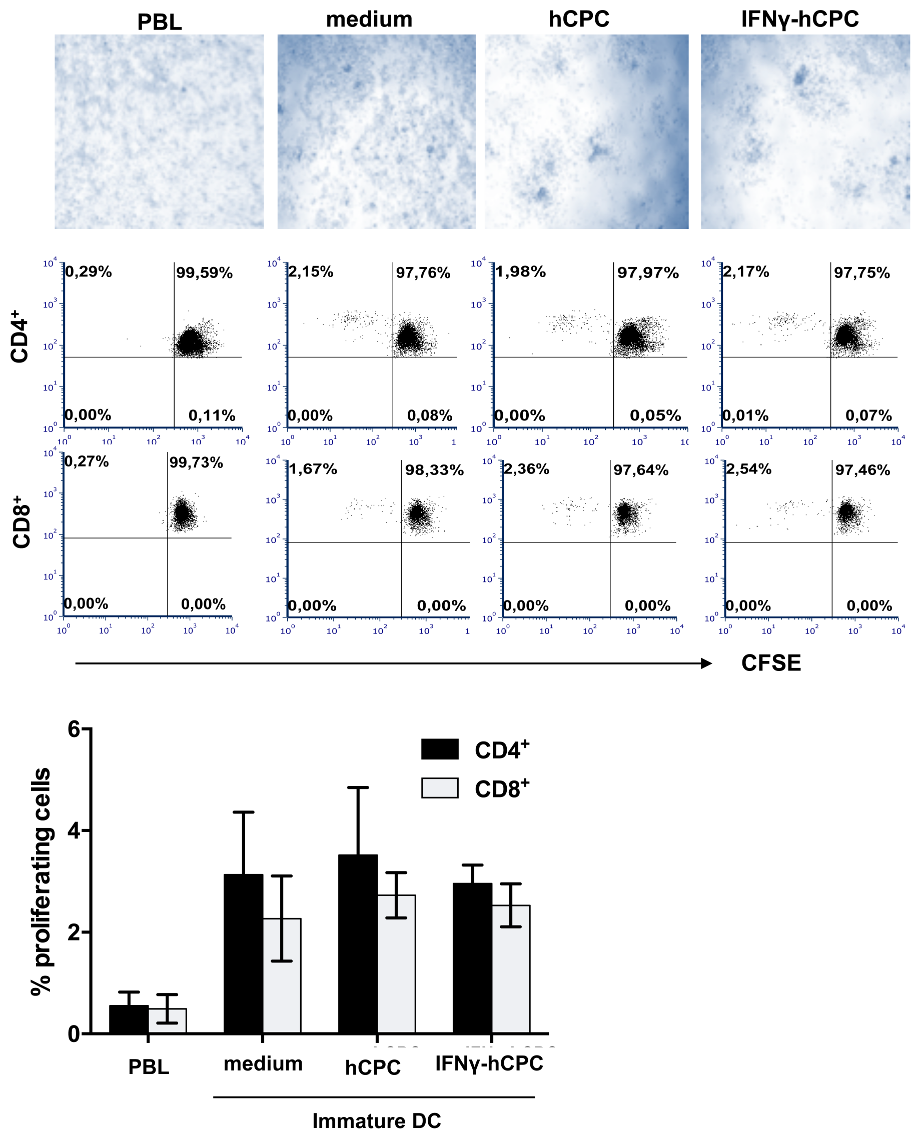

iDC generated in the absence (medium) or presence of hCPC (hCPC) or IFNγ-hCPC (IFNγ-hCPC) were used to stimulate the proliferation of allogeneic CFSE-labeled PBL at a PBL/DC ratio of 5:1 (upper panel). The percentage of $CD4^+$ and $CD8^+$ proliferating T-cells was determined by flow cytometry. Representative dot plots from one experiment indicating the percentage of proliferating cells (middle panel), and histograms present mean % proliferating cells ± SEM of triplicates obtained from two independent experiments (lower panel).

Table 1: Antibodies used through out the study

NAME	FLUOROCROME	HOST	CLONE	CATALOG NUMBER	COMPANY
CD45	FITC	MOUSE	HI30	555482	BD
CD14	APC	MOUSE	M5E1	555399	BD
CD1a	PE	MOUSE	HI149	555807	BD
CD16	PE	MOUSE	3G8	555407	BD
CD32	PE	MOUSE	3D3	552884	BD
CD40	PE	MOUSE	5C3	555589	BD
CD80	PE	MOUSE	L307.4	557227	BD
CD86	PE	MOUSE	IT2.2	555665	BD
CD83	PE	MOUSE	BH15e	556855	BD
CD163	PE	MOUSE	GHI/61	556018	BD
CD206	PE	MOUSE	19.2	555954	BD
TLR2(CD282)	PE	MOUSE	11G7	565349	BD
HLA-II	PE	MOUSE	WR18	MA1-80680	ebiosciences
CD64	PE	MOUSE	10.1	CD6404	Miltenyi Biotech
CD3	APC	MOUSE	SK7	345767	BD
CD4	PACIFIC BLUE	MOUSE	RPA-T4	558116	BD
CD8	APC	MOUSE	SK1	560179	BD
CD25	PE	MOUSE	4E3	130-091-024	Miltenyi Biotech
